# Shade tolerance in Swarnaprabha rice is associated with higher rate of panicle emergence and positively regulated by genes of ethylene and cytokinin pathway

**DOI:** 10.1038/s41598-019-43096-8

**Published:** 2019-05-02

**Authors:** Madhusmita Panigrahy, Aman Ranga, Jyotirmayee Das, Kishore C. S. Panigrahi

**Affiliations:** 1 National Institute of Science Education and Research, Homi Bhabha National Institute (HBNI), 752050 Khurda, India; 2 Orissa University Agriculture and Technology, Bhubaneswar, Odisha 751003 India

**Keywords:** Abiotic, Light stress

## Abstract

This study identifies characteristics of seedling, mature plant phenotypes, changes at genetic and genomic level associated with Swarnaprabha (SP) rice grown under prolonged shade and compared with Nagina 22 (N22). Coleoptile length under low red/far-red was intermediate between that in dark and red light in a 7-days growth frame. Whereas, highest rootlet number was discriminating in seedlings grown for 28 days in hydroponics. In shade, SP and N22 both showed several tolerant mature plant phenotypes, except the panicle length, yield per plant and % grain filling, which were higher in SP. Percentage decrease in yield / plant in shade showed significant positive correlation with increase in NDVI, decrease in panicle length and % grain filling (p ≤ 0.01). Rate of panicle emergence in shade was higher in SP than N22. Expression patterns of *PHYTOCHROME INTERACTING FACTOR LIKE-13* and *PHYTOCHROME B* were contrasting in SP and N22 seedlings under continuous red or red/far-red. Microarray analysis revealed the up-regulation of most of the ethylene and cytokinin pathway genes in shade grown panicles of SP. Significant up-regulation of *ETHYLENE RESPONSE ELEMENT BINDING PROTEIN-2*, *MOTHER OF FLOWERING TIME 1*, and *SHORT PANICLE1* genes in shade grown panicles of SP could explain its sustainable higher yield in shade.

## Introduction

Crop yield is affected by low R/FR ratio of incident light, which is the characteristic of canopy or shade. Light signals due to shade and neighboring vegetation cause large changes in plant form and function including elongated internodes, more erected leaves, higher chlorophyll content and early flowering. Light signals perceived by the canopy is affected from the irradiance of canopy architecture, intercept of photosynthetically active radiation affect yield components like flowering time, grain number, yield per plant^1–3^. Although plant responses to R/FR are critical for shade avoidance, it can be detrimental for the yield as the investment of resources into organs that increase shade avoidance (e.g. longer stems or leaf sheaths) reduces the allocation of resources to harvestable organs (e.g. grains and leaves). Light signals in shade are predominantly perceived by phytochromes and cryptochromes. Shade avoidance responses are known to be primarily regulated by Phytochrome B (PHY B) and its downstream components *PHYTOCHROME INTERACTING FACTOR* (*PIF*) *4* and *PIF5*^1^. There are few traits such as chlorophyll synthesis^1^, chloroplast movement^4^, stomatal opening^5^, auxin signaling^6^, internode length^7^, spike and stem growth^8^ that were shown to be regulated by PHYs and their downstream components. Moreover, several yield related traits such as plant architecture^9^, grain yield^9^, plant height^10^ were known to be affected by the overexpression of PHYs. Several genes involved in sensing the photoperiod such as *GRAIN NUMBER*, *PLANT HEIGHT AND HEADING DATE 7*, *PHOTOSENSITIVITY* 13 and *HEADING DATE 1* were shown to affect various yield related parameters such as dry weight, grain yield, filled grains, primary and secondary branches in rice^11^. However, details of light perception, altered clock regulations and the components of yield pathway that are affected in low R/FR light are not fully known. Several reports have shown shade tolerance properties of the rice line Swarnaprabha (SP) such as low light irradiance tolerance, sustainable yield in shade^12–14^. However, detailed molecular mechanisms of shade tolerance in SP are far from being understood. Knowing these mechanisms will help for sustainable crop yield under canopy shade or unpredictable cloudy climate conditions.

The aim of this study was to unravel several requisite information for growing under low light or low R/FR condition. Answers were sought to (i). The duration of rice (short/medium/long) that is best suited for growing in shade i.e. at low R/FR, (ii). The seedling or mature plant phenotype that could be taken as the characteristic for obtaining low R/FR tolerance in field condition, (iii). Expression of genes that are known to be linked to yield and related attributes under low-light, and (iv) The genomic changes underlying sustainable yield of Swarnaprabha were attempted. For this purpose, three different durations of rice varieties were selected including Nagina 22 (N22) as short duration, KMR3 as medium duration and Swarna as long duration rice and compared with Swarnaprabha (short duration) which is the most studied shade tolerant rice variety. From each duration type, one introgression line (IL) or mutant (NH) was included on the basis of their yield (higher or lower than their respective controls) in field condition^12^. Seedling phenotype screening was considered to include novel variability from diverse sources. *NH686* and 192S were selected as high yielding lines than N22 and Swarna respectively, whereas 399 was selected as low yielding line than KMR3.

## Results

### Seedling phenotype

**Figure 1:**
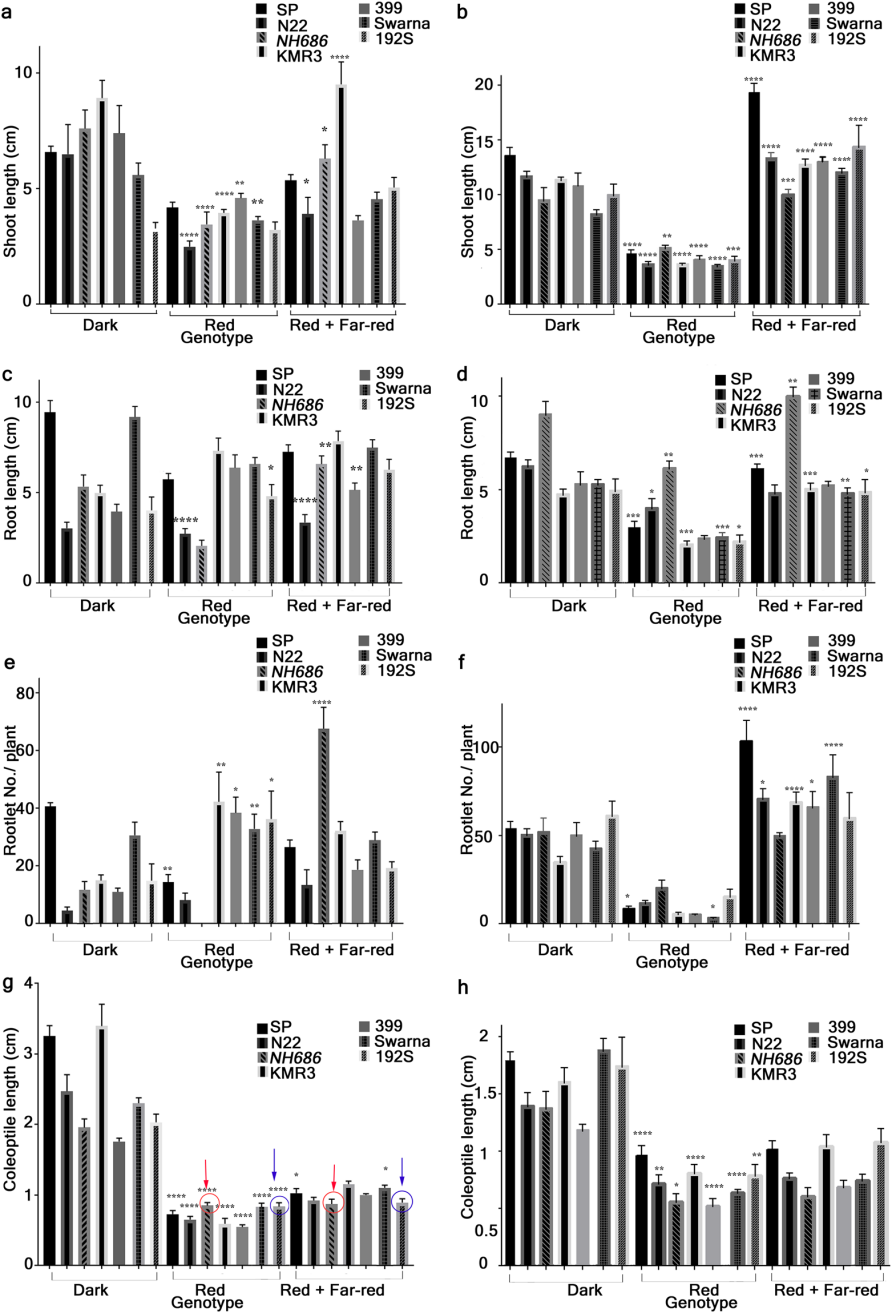
Seedling phenotype analysis in low Red/far-red (R/FR). Seven genotypes (SP, N22, KMR3, Swarna, the mutant of N22 *NH686*, the IL of KMR3 399 and the IL of Swarna 192S) were analyzed for seedling phenotypes such as shoot length (SL), root length (RL), rootlet number (RN) and coleoptile length (CL). (**a**,**c**,**e**) Seedlings were grown for 7 days on petriplates or (**b**,**d**,**f**) in hydroponic medium till 28 days in Dark, continuous Red (cR) or low cR/FR. Each data value is a mean of 55–60 readings obtained from two biological replicate experiments. Significance was determined by comparing phenotype of Dark with Red grown seedlings and the Red with the Far-red grown seedlings. Significance values obtained from one-way Anova are represented as *P ≤ 0.05; **P ≤ 0.01; ***P ≤ 0.001; ****P ≤ 0.0001. (**e**) *NH686* and 192S seedlings having similar coleoptile length in cR and cR/FR are marked with RED and BLUE circle and arrows respectively.

Seedlings irradiated with either continuous red (cR), under low red/far-red (cR/FR) or kept in dark (D) were evaluated for shoot length, root length, rootlet number or coleoptile length (Fig. 1 and Supplementary Fig. 1). The analyses at two time points i.e. 7 days on petriplates or 25 days in hydroponic medium would not only answer the effect of cR/FR on the seedling phenotype at these two stages but also it would dissect the phenotype caused due to direct exposure of the roots (on petriplates) to light and nutrient supplement in hydroponic. On petriplates, shoot length (SL) of most of the lines were significantly less under cR (Fig. 1a). In addition to this characteristic, the SL growth inhibition under cR, significantly longer SL under cR/FR was also observed in all the lines when grown in hydroponic medium (Fig. 1b). Here, it was prompting to interpret that the seedlings grown in hydroponics for longer period represent clearer platform for identifying the characteristic seedlings phenotype while screening under cR/FR. While the root length (RL) of the lines did not have any characteristic elongation/inhibition from D to cR or from cR to cR/FR on petriplates (Fig. 1c), the RL of most of the lines grown in hydroponics showed significantly shorter RL under cR compared to D and also longer under cR/FR compared to cR (Fig. 1d). The rootlet number (RN) of the rice lines on petriplates was significantly higher under cR than the D (Fig. 1e), with the only exception in SP which had a smaller number of rootlets under cR than D and *NH686*, which had no rootlets under cR. Number of rootlets of the seedlings grown in hydroponics was significantly higher under cR/FR than that under cR with the highest rootlet number was observed in SP (Fig. 1f). Clearly, the RN of the seedlings under cR/FR in hydroponics can be taken as distinguishing phenotype. Coleoptile length (CL) of seedlings grown under cR on petriplates or hydroponics was significantly less than D (Fig. 1g,h). The CL in mutant *NH686* and the IL 192S under cR was similar to that under cR/FR indicating higher sensitivity to low R/FR (Fig. 1g, circles with arrows). Results of seedling phenotype analyses showed that seedlings have different growth characteristics when grown on petriplates or in hydroponics. Higher SL, RL and RN could be taken as distinct phenotype under cR/FR when grown in hydroponics, whereas on petriplates seedlings grown under cR had distinctly short SL and CL.

The mutant *NH686* and IL 192S were selected for further analysis. Selection of these lines was the result of screening of 13 ILs and mutants (data not shown) along with their controls and SP at seedlings stage for having either similar phenotype like SP or for having altered phenotype than their respective controls (data not shown). These two lines were also high yielding under sun which significantly out-performed their controls^12^. The low yielding 399 was taken for study to answer if there were any correlation of yield in field condition with any of the mature plant phenotype or yield under shade.

### Mature plant phenotype

**Figure 2:**
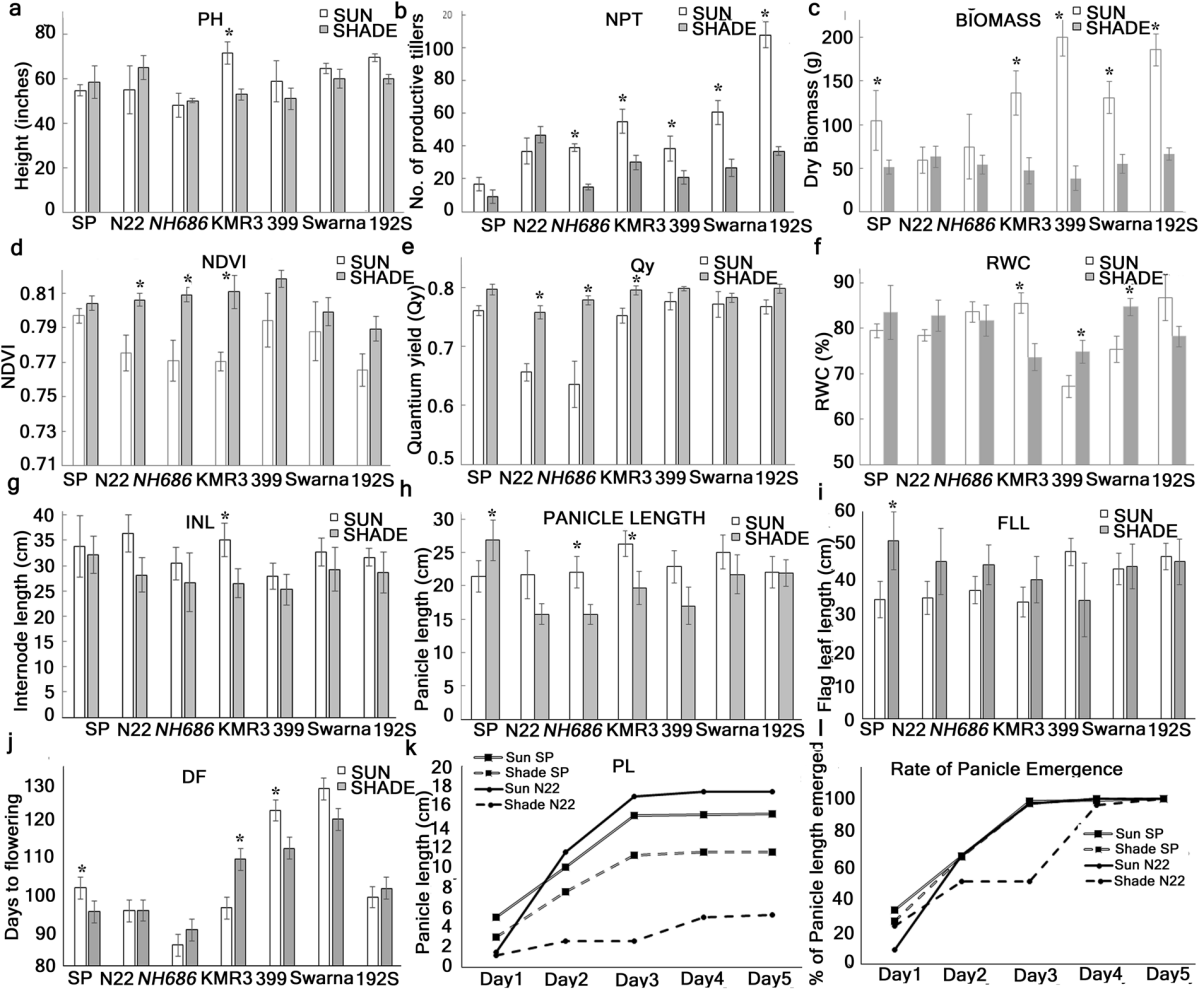
Mature plant phenotype of 7 rice genotypes in continuous shade. Plants at 55 days after showing (DAS) were kept in shade. Mature plant phenotypes were recorded 15 days after day to flowering (DF). Each data value is a mean of 40–45 plants grown in two seasons. (**a**) PH: length of the tallest tiller from the soil surface, (**b**) NPT: total number of productive tillers, (**c**) Biomass: dry weight of above ground biomass after harvest, (**d**) NDVI: normalized difference vegetation index of the 2^nd^ or 3^rd^ leaf from top, (**e**) Qy: quantum yield of the flag leaf, (**f**) % RWC: relative water content of the 2^nd^ or 3^rd^ leaf from top, (**g**) INL: length of the 1^st^ internode from top, (**h**) Panicle length: length of the panicle after harvest, (**i**) FLL: length of the flag leaf, (**j**) DF: days to flowering. (**k**,**l**) For calculating rate of panicle emergence, length of panicle emergence from the 1^st^ day it is visibly emerging out from the main shoot was measured till 1^st^ 5 days. This was performed in sun and shade grown panicles of SP and N22. *Represents significant differences at p < 0.05.

Mature plant parameters which are known to respond to low light may provide a strong indication of tolerance to continuous shade among the tested lines. In response to continuous shade, SP and N22 showed increase in plant height and KMR3 showed maximum decrease of about 34.9% (Fig. 2a and Supplementary Table 2A). All the lines tested showed decrease in number of productive tillers (NPT) with maximum decreased was observed in 192S (Fig. 2b and Supplementary Table 2A). Above ground dry biomass was drastically reduced in all the lines tested in continuous shade except N22 which showed 6.7% increase (Fig. 2c and Supplementary Table 2A). NDVI (normalized density vegetation index) and quantum yield (Qy) showed increase in all the lines in response to continuous shade with maximum increase was observed in *NH686* (Fig. 2d,e). Relative water content (% RWC) in response to shade showed variation among the tested lines with increase in 399, Swarna and decrease in KMR3 and 192S. SP, N22 and *NH686* showed neglible changes in RWC in response to continuous shade (Fig. 2f). The 1^st^ internode (INL) has been reported to have growth elongation in response to shade^7^. In the present study, all the lines tested showed insignificant decrease in INL in response to continuous shade (Fig. 2g). We further tested the length of the panicle in response to shade. All the lines showed decrease in panicle length (PL) except SP, which showed significant increase of about 25% in shade (Fig. 2h and Supplementary Table 2A). Flag leaf length (FLL) has been shown to increase under low light^11^. FLL in our study was increased significantly in SP and decreased in 399 (Fig. 2i).

From the study of all these parameters, it was indicated that under continuous shade, most of the mature plant phenotypes such as PH, NPT, NDVI, QY and FLL which were known as parameters that respond during shade avoidance of rice plant, were showing similar response for most of the lines tested, hence could not be taken as screening parameters for obtaining a shade tolerant plant. N22 and *NH686* showed many of the mature phenotypes such as PH, biomass, NDVI, QY promising under shade indicating shade tolerance, except the PL in these two lines, which showed significant decrease under shade. Among all the mature plant phenotype tested, only the PL was identified as the discriminating parameter between SP and other lines in this study.

### Yield attributes

To understand the effect of shade on yield attributes, days to flowering (DF), 100-grain weight (100-Gwt), % grain filling (GF), grain weight/panicle (Gwt/pa) and yield per plant (Yi/pl) were studied in the rice genotypes and their harvest after continuous shade (Table 1). DF was reduced indicating early flowering under continuous shade in case of the lines SP, 399 and Swarna, whereas delayed flowering was observed in KMR3 and 192S (Fig. 2j and Table 1). 100-Gwt of the shade grown plants was decreased in 5 out of 7 lines tested, with minimum decrease of 7.8% in *NH686* followed by SP with 10.7% decrease (Supplementary Table 1). The 100-Gwt of the lines KMR3 and 192S had marginal increase under shade. % GF decreased in the shade grown plants in most of the lines except SP and 192S, with SP having maximum increase of about 40%. Gwt/pa from the shade grown plants decreased in comparison with that of sun in all the lines except SP and Swarna, with the highest increase of 16.5% in SP (Supplementary Table 1). Yi/pl from the shade grown plants showed decrease in all the lines with the least decrease of 17.3% in SP (Supplementary Table 1).

**Table 1:** Yield and related attributes from 7 rice lines grown in shade.

	DF		100-Gwt		Yield/Pl		Gwt/Panicle		%GF	
	SUN	SHADE	SUN	SHADE	SUN	SHADE	SUN	SHADE	SUN	SHADE
SP	**101**.**6** ± **5**.**8**	**95** ± **5**.**2**	**2**.**55** ± **0**.**57**	**2**.**28** ± **0**.**11**	23.64 ± 7.16	19.53 ± 1.81	1.52 ± 0.75	1.77 ± 0.002	57.45 ± 13.55	80.56 ± 18.99
N22	95.2 ± 4.6	95.33 ± 4.2	**1**.**84** ± **0**.**08**	**1**.**32** ± **0**.**05**	**35**.**17** ± **0**.**79**	**9**.**08** ± **0**.**68**	**1**.**47** ± **0**.**64**	**0**.**40** ± **0**.**06**	81.20 ± 0.55	69.14 ± 14.73
*NH686*	85.8 ± 4.8	90 ± 4.5	**1**.**80** ± **0**.**05**	**1**.**66** ± **0**.**02**	**49**.**50** ± **3**.**12**	**12**.**24** ± **4**.**4**	**1**.**48** ± **0**.**08**	**0**.**69** ± **0**.**41**	**81**.**79** ± **5**	**75**.**15** ± **0**.**03**
KMR3	**96** ± **5**.**9**	**109**.**5** ± **5**.**3**	1.94 ± 0.15	1.99 ± 0.06	**39**.**70** ± **14**.**99**	**12**.**05** ± **2**.**5**	**0**.**78** ± **0**.**19**	**0**.**40** ± **0**.**07**	56.81 ± 14.94	56.41 ± 6.84
399	**123** ± **5**.**2**	**112**.**5** ± **5**.**6**	**1**.**96** ± **0**.**13**	**1**.**53** ± **0**.**01**	**37**.**73** ± **9**.**98**	**4**.**47** ± **2**.**46**	**0**.**81** ± **0**.**25**	**0**.**27** ± **0**.**13**	58.82 ± 17.1	58.20 ± 7.12
Swarna	**129** ± **6**.**5**	**120**.**5** ± **6**.**2**	**1**.**94** ± **0**.**15**	**1**.**52** ± **0**.**06**	**61**.**62** ± **1**.**81**	**31**.**20** ± **8**.**57**	1.23 ± 0.16	1.36 ± 0.27	77.51 ± 4.03	55.45 ± 25.09
192S	99 ± 6.2	101.5 ± 6.8	1.69 ± 0.21	1.76 ± 0.07	**30.97** ± **18**.**29**	**20**.**65** ± **3**.**56**	0.71 ± 0.26	0.50 ± 0.25	46.96 ± 5.55	55.43 ± 8.27

DF: Days to flowering from the day of sowing, 100-Gwt: weight (grams) of 100 filled grains, Gwt/Panicle: Total weight of filled grains from per panicle, Yield/pl: yield per plant, % GF: percentage grain filling. Each data value is a mean of 30 readings obtained from 2 biological replicate experiments. Statistically significant differences between the means at p < 0.05 are indicated in bold letters.

Percentage decrease in yield/plant from sun to shade in the tested lines in this study showed significant positive correlation with increase in NDVI index, decrease in panicle length and % grain filling at p ≤ 0.01 (Supplementary Table 2).

### Biochemical characteristics

**Figure 3:**
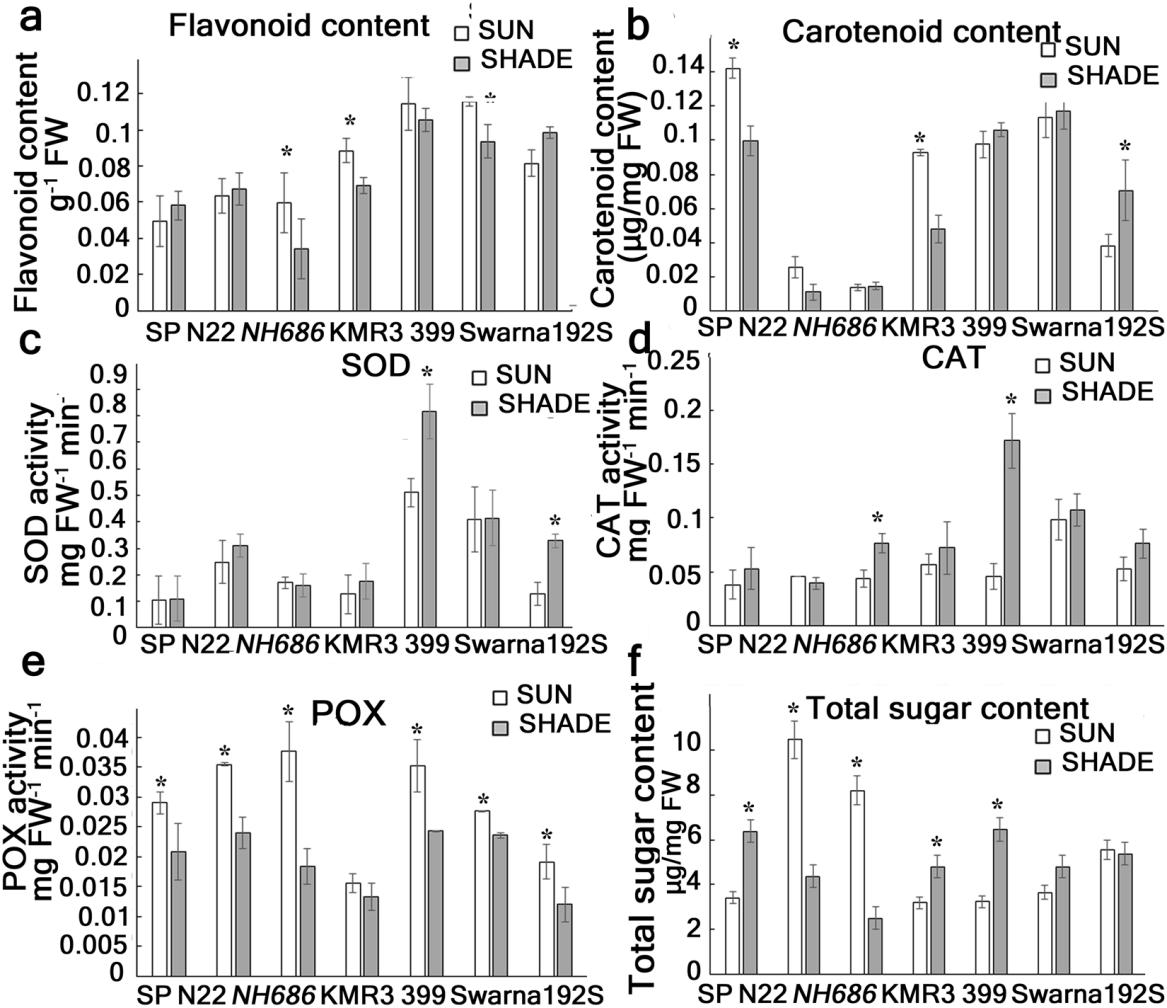
Biochemical characteristics of the mature plant leaf samples from plants grown in shade. All biochemical analyses were performed from flag leaves at 15 days after DF. Each data value is a mean of 20 readings obtained from 2 biological replicate experiments. *Represent significant differences at p < 0.05. SOD: superoxide dismutase activity, CAT: catalase activity, POX: peroxidase activity.

A 75% cutoff of the day light imposed after late vegetative period could act as low light stress in our study, as we observed several stress responses in our genotypes (Fig. 3). Flavonoid accumulation was significantly reduced in *NH686*, KMR3 and Swarna whereas in SP and N22 the accumulation showed neglible change from sun to shade (Fig. 3a). Carotenoids have always been related to temperature stress. Presently, N22 and its mutant had least total carotenoid in sun as well as shade, whereas SP and KMR3 showed significant (29.8% and 48.5% respectively) decrease in carotenoid content in shade grown plants (Fig. 3b, Supplementary Table 1). To understand the enzymatic activity for detoxification of the reactive oxygen species generated, assays of superoxide dismutase (SOD), catalase (CAT) and peroxidase (POX) were performed. The SOD activity in SP the flag leaves in sun and shade was significantly less than other genotypes. The ILs 399 and 192S showed significant increase in SOD activity (37.5% and 60.8% respectively) under shade compared to sun (Fig. 2c and Supplementary Table 1). CAT activity showed neglible increase in shade in SP, N22 and Swarna, whereas highest increase was observed in 399 (73.2%) (Fig. 3d and Supplementary Table 1). POX activity was decreased in all the lines tested except KMR3 (Fig. 3e, Supplementary Table 1). Under prolonged shade, total sugar content showed variation in different lines tested. Significant increase in total sugar content in shade was observed in SP, KMR3 and 399 (46.5%, 49.9% and 23.9% respectively), whereas significant decrease in sugar content was observed in N22 and its mutant (Fig. 3f and Supplementary Table 1).

These effects of continuous shade on different biochemical parameters indicated that most of the biochemical parameters tested here did not correlate with high yield among the tested lines. However, the decreased SOD activity in shade can be considered as a distinguishing characteristic for shade tolerance, as it was significantly less SP in sun and shade than that of the other lines. Moreover, the low yielding line 399 showed highest increase in SOD and CAT as well as highest decrease in POX indicated for having opposite response to SP in shade.

### Total carbon, phosphorus and nitrogen

**Figure 4:**
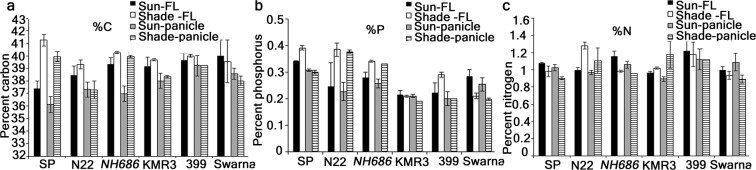
Estimation of carbon, phosphorus and nitrogen in the flag leaf and panicle samples. Flag leaf and panicle samples at final stages of grain filling (~15–21 days after final panicle emergence) were processed for estimation of carbon (C), phosphorus (P), nitrogen (N) as described in Materials and Methods. Each data point is a mean of 12 readings obtained from samples grown in 2 seasons.

Partitioning of C, N and P in the flag leaves and panicles in the later stages of grain filling may provide meaningful clues of efficient mobilization of nutrients and photoassimilates from the flag leaves to the grains. In SP, efficient mobilization of C to the panicles was observed in shade compared to sun, as significantly higher % of C was observed in shade in the FL and panicles (Fig. 4). However, % P and % N were not higher in shade compared to sun. % P for N22 and *NH686* showed similar trend with % C in SP indicating higher % of P and N in their FL and panicles in shade than in the sun. % C was also higher in *NH686* panicles in shade than sun. Flag leaves and panicles of KMR3 had less % P and % C partitioned in shade than sun except the % N in panicles in shade which was higher than that of sun. In shade, all the 3 nutrients % were less in the panicles of 399 compared to that of sun. In Swarna, the % of all the 3 nutrients were less in both the flag leaves and panicles in shade than sun. These results indicated that for sustainable yield in shade, higher % C is essential. Moreover, low yield in sun might lead to low yield in shade associated with less % of P, N and C partitioning in the panicles in shade.

### Rate of panicle emergence

N22 showed several mature plant phenotypes similar to a shade tolerant plant starting from total carotenoid, flavonoid accumulation to maximum increase in height, NPT, above ground biomass and maintained % RWC. However, it was negatively associated with the yield attributes such as decrease in PL, INL, Gwt, Gwt/pa and Yi/pl. Hence, the most characteristic feature that deviated between SP and other lines, the PL, was selected for further study between N22 and SP in sun and shade (Fig. 2k,l). To this end, length of emerging panicles was measured daily from the 1^st^ day it was visible as emerging out from the main shoot till the 1^st^ five days of emergence. The PL varied significantly from the 1^st^ day of emergence between SP and N22 in both sun and shade (Fig. 2k). However, rate of emergence was significantly less in N22 than SP in shade on the 2^nd^ day and this slower rate persisted till 4^th^ day. The rate of emergence as well as the PL varied to the maximum on 3^rd^ day of emergence in shade, while N22 had ~50% rate of emergence, SP had rate of emergence of about 97.3% (Fig. 2l).

### Expression of *phytochrome B* and *phytochrome interacting factors like* (*PILs*) transcripts in low R/FR

**Figure 5:**
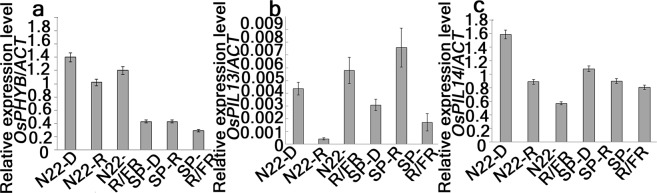
Gene expression of *PHYB*, *PIL13* and *PIL14* in SP and N22 seedlings. Gene expression using (**a**,**c**) semi-quantitative RT PCR and (**b**) real-time QPCR were performed from 7-days-old seedlings of SP and N22 irradiated with either Red (R), low R/FR (R/FR) or in continuous dark (**d**). (**a**,**c**) Relative expression level was calculated using Image lab software (Version 6.0.0, 2017, Bio-rad Laboratories) by taking SP-D, N22-D as reference bands for calculation of intensities of R and R/FR of respective genotypes.

PHYB has been reported to be the major photoreceptor for mediating light enriched with low R/FR with its downstream interacting partner PIF4 was reported as the master player of SAS (shade avoidance syndrome) responses^15^. However PIL13 and PIL14 were reported to be more identical to *Arabidopsis* PIF4 sequentially^16^. Hence, the expressions of *PHYB*, *PIL13*, *PIL14* were analyzed in continuous dark (D), red (cR) and low R/FR (cR/FR) in N22 and SP (Fig. 5). Transcript levels of PHYB was maintained under cR, whereas reduced under cR/FR compared to D in SP. On the contrary, reverse trend of PHYB transcript level including reduced expressions in cR than D and increased expressions in R/FR than cR was observed in N22 (Fig. 5a). Significant up-regulation of Os*PIL13* and down regulation of Os*PIL14* in SP under cR was observed (Fig. 5b,c). Under cR/FR, transcripts of the 2 *PIL*s in study had least expression levels in SP (Fig. 5b,c). In N22, expression pattern of *PIL14* was similar to that of SP, though with different relative values (Fig. 5c). However, *PIL13* expression pattern in N22 was reverse with that of SP in cR and cR/FR compared to D (Fig. 5b).

### Genome-wide expression analysis in SP and comparative analysis of genes in N22 under prolonged shade

**Figure 6:**
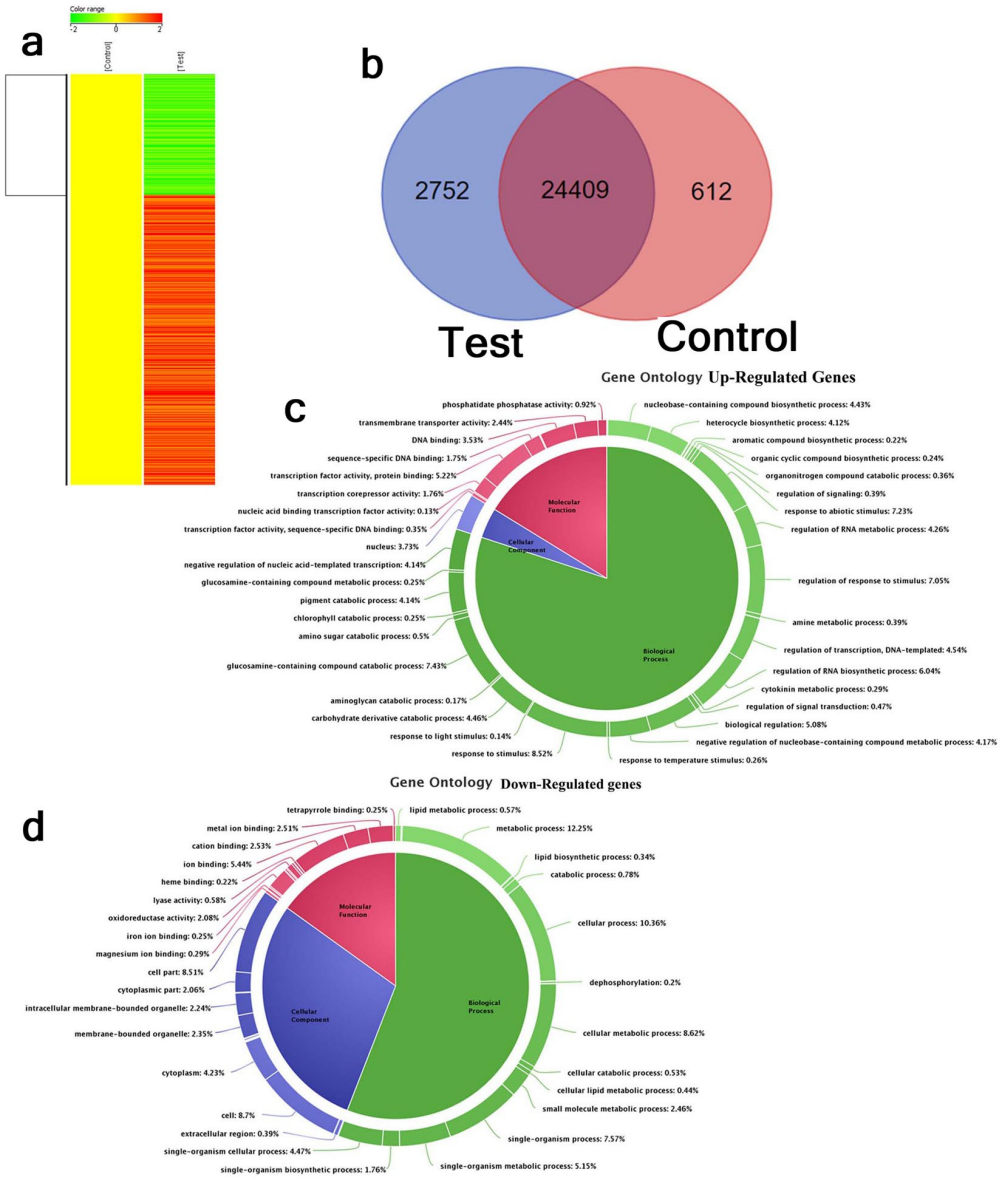
Genome-wide expression analysis using microarray. The panicles of SP grown in sun or shade were sampled on the day of final emergence from the main shoot. (**a**) Heat map of the differentially regulated genes. (**b**) Venn diagram showing number of genes detected in control and test samples. (**c**,**d**) Pie- diagram showing Gene-ontology of up and down- regulated genes respectively.

**Figure 7:**
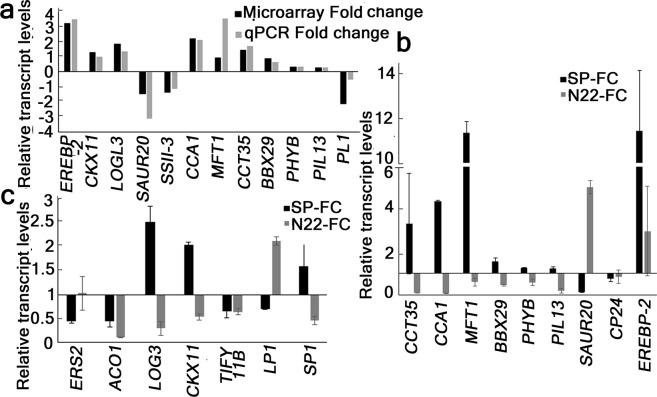
Validation of microarray results and comparative analysis of gene expression between SP and N22 using real-time PCR. (**a**) Coherence of the expression pattern for 12 genes in microarray and real-time PCR. Expression levels were compared in sun and shade grown panicle samples of SP with SP-Sun expression as controls. The relative transcript levels were shown as Log2 fold change. (**b**,**c**) Relative transcript levels from real-time PCR from panicle samples of SP and N22 grown in sun or shade. (**b**) Relative transcript levels of genes common in light and yield related pathway. (**c**) Transcript levels of genes common in hormone and yield related pathway. (**b**,**c**) Comparative presentation of fold change of selected genes between sun and shade grown samples in their respective genotypes of SP and N22. For each fold change calculation, the expression level in sun sample of the respective genotype was taken as control. All expression levels were normalized first to their respective *ACTIN* expression levels. Each real-time data is mean of 2 biological replicate experiments performed in triplicates and the error bar represents the standard error of mean.

To understand global expression profiles of different genes, microarray experiment was performed from sun and shade grown SP panicles (Fig. 6). Total 41714 probe sets were used for detection, which showed 25022 detections in the control samples and 27162 detections in the test samples (Fig. 6b). At this stage of analysis, 24409 probe sets were commonly detected in both control samples and test samples. While 2752 probe sets were uniquely detected in the test samples, 612 probe sets were unique to the control samples (Fig. 6b). Differential gene expression (DEG) analysis resulted a total of 2110 genes up-regulated and 876 genes down-regulated in the test compared to the control. Among the up- and down- DEGs, 12 genes were selected for the validation of microarray results. Coherence of the real-time PCR results with the (Fold change) FC of the microarray (Fig. 7a) indicated the significance and biological relevance of the results. Categorizing according to GO annotations resulted 55.88% of genes in biological process, 29.01% of genes in cellular component and 15.05% of genes in the molecular function category among the down regulated genes. Similarly among the up-regulated genes, 80.03% of genes in biological process, 3.73% of genes in cellular component and 16.22% of genes were involved in the molecular function (Fig. 6c,d). Further pathway analysis using KEGG pathway and GO annotation resulted 615 genes in metabolic pathway, 99 genes in secondary metabolite biosynthesis and metabolism, 51 genes in cellular process and 191 genes in carbohydrate metabolism and photosynthetic processes. Further selection was based on genes involved in 9 different hormone pathways, response to different abiotic stresses, regulation of circadian clock, light signaling pathway and yield related attributes (Table 2). Up-regulated genes primarily included ethylene pathway, cytokinin pathway, ABA pathway, clock regulations and light signaling. More than 2/3^rd^ genes identified in the abiotic stress and auxin pathway were up-regulated, whereas nearly 1/3^rd^ were down-regulated. Among the down regulated genes, several sugar transporters, GA dioxygenases, starch synthase, photosystem II PSBS2, seed storage protein, senescence specific cysteine protease were the major category involved (Table 2).

**Table 2:** List of gene from microarray analysis arranged according to different pathways.

Gene ID	Gene Name	p-value	FC	UP/Down
**(BOX-1) Ethylene**				
Os03t0860100-01	EREBP-2, ERF83	0.3832	3.190	Up
Os08t0474000-01	ERF104	0.6535	1.822	UP
Os02t0654700-01	ERF91	0.1975	1.650	UP
Os02t0521100-01	ERF107	0.2824	1.647	UP
Os04t0546800-01	ERF93	0.2830	1.424	UP
Os04t0398000-02	ERF101	0.0116	1.292	UP
Os09t0451000-01	ACO1,OsACO1, ACO	0.0045	1.282	UP
Os09t0451000-01	ACO2	0.0045	1.282	UP
Os07t0685700-01	EIL2, OsEIL2	0.3446	1.217	UP
Os05t0155200-01	ERS2	0.0430	1.152	UP
Os07t0410700-01	ERF86	0.4836	1.125	UP
Os04t0529100-01	ERF45	0.2796	1.120	UP
Os12t0168100-01	ERF124	0.5890	1.066	UP
Os05t0549800-01	AP2/EREBP96	0.1626	0.978	UP
Os02t0676800-01	ERF20	0.6976	0.933	UP
Os03t0183200-01	ERF69	0.2799	0.867	UP
Os09t0490200-02	EIL3	0.0086	0.863	UP
Os09t0490200-02	EIL3	0.0086	0.860	up
Os11t0129700-01	AP2/EREBP131	0.0305	−1.065	Down
**(BOX-2) Auxin**				
Os01t0919800-01	PIN5A	0.1089	2.260	UP
Os04t0617050-01	SAUR21	0.0683	2.244	UP
Os01t0257300-01	RAA1	0.3321	2.074	UP
Os01t0888800-01	ARGOS	0.4012	1.904	UP
Os01t0785400-01	GH3-1	0.1521	1.582	UP
Os01t0741900-01	IAA6	0.2434	1.231	UP
Os01t0741900-01	IAA26	0.2434	1.230	Up
Os04t0519700-01	ARF10	0.1293	−0.848	Down
Os05t0515400-01	OsARF14, OsETT4	0.0531	−1.263	Down
Os04t0608300-01	SAUR20	0.1313	−1.440	Down
Os04t0690600-01	ARF13	0.1301	−1.611	Down
Os05t0576900-01	PIN10B	0.0753	−1.956	Down
**(BOX-3) Cytokinin**				
Os03t0224200-01	RR21	0.1304	0.830	UP
Os01t0940000-01	CKX4, OsSCRM	0.3475	2.039	UP
Os03t0109300-01	LOGL3	0.2887	1.852	UP
Os05t0521300-02	PHP3	0.3054	1.751	UP
Os01t0588900-01	LOG1	0.1521	1.577	UP
Os11t0143300-01	OsRR9	0.0019	1.460	up
Os08t0460600-01	CKX11	0.1433	1.308	UP
Os01t0775400-01	CKX5	0.0788	0.940	UP
**(BOX-4) Jasmonic acid**				
Os03t0402800-02	TIFY10A	0.1548	2.094	UP
Os03t0181100-01	TIFY11B, OsJAZ4	0.4373	1.740	up
Os04t0395800-01	TIFY9	0.5121	1.416	UP
Os03t0180900-01	TIFY11C	0.5111	1.304	UP
Os10t0392400-01	TIFY11D	0.4468	1.296	UP
Os06t0314600-01	JMT1	0.2354	−1.366	Down
**(BOX-5) Photosynthesis**				
Os04t0457000-01	CP24, Lhcb6	0.0754	1.915	UP
Os01t0700100-01	SWEET2B	0.4279	1.499	UP
Os09t0532000-01	SGR, sgr(t), sgr, OsSGR	0.0763	1.451	UP
Os03t0341300-01	SWEET16	0.2247	1.368	UP
Os09t0325700	PP2C1	0.3364	1.290	up
Os07t0544800-01	OEE3	0.0549	1.007	UP
Os12t0190200-01	PsbP	0.1003	0.958	UP
Os06t0603600-02	OsSPX1	0.0669	0.944	UP
Os12t0564400-02	PsbP	0.0793	0.886	UP
Os12t0476200-01	SWEET13	0.2999	0.875	UP
Os05t0549100-01	STN7	0.3179	0.818	UP
Os01t0934400-01	lumenal PsbP	0.3591	0.824	UP
Os04t0690800-01	PSBS2	0.0455	−0.857	Down
Os04t0452700-01	OsMST1	0.1664	−1.257	Down
Os06t0229800-01	alk, SSII-3, OsSSIIa, OsSSII-3	0.1012	−1.369	Down
Os01t0567600-00	OsMST7	0.1690	−1.788	Down
**(BOX-6) Abiotic Stress**				
Os02t0758000-01	HSP24.1	0.3806	1.716	UP
Os05t0122700-01	OsRCI2-7	0.0055	1.763	UP
Os02t0758000-01	HSP24.1	0.3806	1.716	UP
Os02t0758000-01	HSP24.1	0.3806	1.716	UP
Os06t0553100-01	HSFC2B	0.3862	1.570	UP
Os04t0568700-00	HSFB2A	0.3818	1.560	UP
Os03t0272300-02	OsSDIR1, SDIR1	0.3309	1.554	UP
Os09t0526600-03	HSFB2C	0.4015	1.461	UP
Os12t0623600-01	2-aminoethanethiol dioxygenase	0.0154	1.372	UP
Os03t0267000-00	HSP18.0	0.5043	1.342	UP
Os07t0129200-01	PR1A, OsPR1a	0.0526	1.173	UP
Os10t0392600-01	OsSPX3	0.0672	1.169	UP
Os02t0782500-02	HSP24.1	0.4006	1.125	UP
Os01t0382000-01	PR1B	0.1514	1.024	UP
Os08t0546800-01	HSFB2B	0.4441	0.999	UP
Os06t0253100-01	hsp20/alpha	0.5388	0.939	UP
Os01t0279200-02	proteasome subunit	0.1834	0.913	UP
Os02t0285300-01	OsDREPP2, DREPP2	0.0545	0.890	UP
Os01t0758300-02	Osppc3, Osppc2, ppc3, ppc2	0.0961	−0.942	Down
Os01t0733200-01	HSFC1B	0.4520	−1.742	Down
Os01t0701700-01	SAM dependent carboxyl methyltransferase	0.1068	−1.900	Down
**(BOX-7) Clock**				
Os08t0157600-01	CCA1, OsCCA1, OsLHY	0.3155	2.200	UP
Os08t0157600-03	CCA1	0.3471	2.045	UP
**(BOX-8) Light**				
Os02t0606200-01	OsBBX4, OsSTH	0.2175	1.920	UP
Os04t0488400-01	FTL6	0.2958	1.787	UP
Os08t0249000-01	OsBBX25, OsG, OsCCT29	0.1953	1.530	UP
Os12t0117400-01	BTBN22 - Bric-a-Brac	0.1759	1.525	UP
Os10t0560400-01	OsCCT35, OsCMF11, OsH	0.4773	1.455	UP
Os11t0118300-01	BTBN20	0.1222	1.412	UP
Os06t0154200-01	D3, SOL1	0.2779	1.063	UP
Os02t0610500-01	OsCOL4, OsD, OsBBX5	0.329	0.971	UP
Os01t0933500-00	flowering promoting factor-like 1	0.0271	0.939	UP
Os10t0572300-01	Leucine rich protein	0.0774	0.936	UP
Os06t0654900-01	OsBBX20, OsE, OsCCT23	0.1932	0.891	UP
Os06t0498800-01	OsMFT1	0.0746	0.945	UP
Os01t0725800-01	OsWD40-24, OsSPA3/4	0.4535	0.860	UP
Os10t0414700-01	PEP	0.0153	−0.838	Down
Os06t0298200-02	OsBBX19, OsM, DTH2	0.4467	−0.849	Down
**(BOX-9) Yield**				
Os09t0527900-01	OsBBX29, OsDBB1	0.2787	0.868	UP
Os04t0660100-01	OsIBH1	0.2802	0.837	UP

Genes from each pathway were selected on the basis of fold change (FC), not on statistical significance p-value. FC represents the Log2 fold change.

### Regulation of the ethylene pathway genes to optimize the responses in shade

Almost all the genes identified in ethylene signaling in this study were up-regulated indicating that ethylene pathway genes are the major positive regulators for shade tolerance in SP (Table 2, Box-1). The report of ethylene and shade responses sharing major subset of genes has already been demonstrated^17^. Several ethylene responsive transcription factors such as *ERF83*, *ERF104*, *ERF91*, *ERF107*, *ERF93*, *ERF86*, *ERF45*, *ERF124*, *ERF20*, *ERF69* and *ERF101* were significantly up-regulated with high FC in the panicle samples of SP in shade. ERFs have long been known, isolated and were induced under various abiotic stress conditions^18^ too. These AP2 class of transcription factors control various agronomic traits including yield by interplay with many hormone pathways such as JA, ABA^18^ and GA^19^. The up-regulation of these ERFs in this study indicated an interplay of different hormones with ethylene to adapt the plant responses to low light stress. Ethylene responsive element binding protein (*EREBP-2*) was indicated in KEGG database in MAPK signaling and also was shown to regulate internode elongation, tillering and yield by down-regulating a GA-biosynthesis gene^20^. Up-regulation of *EREBP-2* in this study to ~11.2 fold in the shade grown panicles indicated its involvement in regulation of higher yield and elongated internodes in SP in shade. On the contrary, in N22 samples *EREBP-2* was up-regulated to only ~2.9 fold, which might explain the reason of its low yield as compared to SP (Fig. 7b). Ethylene biosynthetic gene *ACO1* was down regulated in both SP and N22 samples in shade (~0.45 and ~0.1 fold respectively), however the down-regulation was higher in N22 (Fig. 7c). Ethylene receptor *ERS2* was down-regulated to ~0.45 fold in SP, whereas nearly unaffected at ~1.02 fold in N22 (Fig. 7b) indicating a contrasting regulation. Recent evidence of increased ethylene content associated with decreased grain filling^21^ supported our results of decreased expression of *ERS2* in SP resulting in increased grain filling than that of N22.

### Regulation of the cytokinin pathway genes in shade tolerance

All the genes involved in cytokinin signaling pathway were up-regulated indicating a major positive role of cytokinin pathway genes in fine tuning shade responses (Table 2, Box-3). Cytokinin retards senescence and promotes greening. Shade grown plants showing higher NDVI index in this study supported the up-regulation of cytokinin pathway. Cytokinin perception and signaling involves two-component signal phosphorelay and histidine phospho-transfer proteins and different response regulators. Up-regulation of histidine phospho-transfer containing protein *PHP3* to ~1.75 fold and A-type response regulator *RR9* to ~1.45 fold (Table 2) supported up-regulation of cytokinin pathway in the shade grown SP plant. Cytokinin oxidase/dehydrogenase (CKX) are responsible for degradation of cytokinins. The up-regulation of *CKX4*, *CKX5* and *CKX11* in the present study to ~2 fold, ~0.9 fold and ~1.3 fold (Table 2) respectively indicated increased feedback regulation, which has already been documented before in case of the CKXs^22^ for homeostatic control of cytokinin activity. Os*CKX11* and Lonely Guy Like *LOGL3* genes were shown in the regulation of inflorescence development, panicle size earlier^23^. Up-regulation of *LOG1* and *LOGL3* to ~1.5 fold (Table 2) and ~2.5 fold (Fig. 7) respectively in the present study correlated to longer panicle length which was observed in SP in contrast to N22, where the *LOGL3* was down-regulated to ~0.29 fold (Fig. 7). Hence, the differential regulation of *LOGL3* could be the possible reason of contrasting panicle length SP and N22 in shade. Contrasting regulation of *CKX11* with ~2.04 fold up-regulation in SP and ~0.53 fold down-regulation in N22 samples supported contrasting role of CKX11 in shade grown panicles of these two genotypes.

### Involvement of auxin pathway for shade tolerance

In the shade grown SP panicles, 2/3^rd^ of the auxin pathway genes were up- and 1/3^rd^ of the same were down-regulated (Table 2, Box-2). The up-regulated auxin pathway genes included several IAAs such as *IAA6* and *IAA26*, auxin responsive promotor family *GH3*, flowering promoting factor *FPF1*, *ARGOS* gene involved in regulating organ size and the gene involved in polar auxin localization during asymmetric division and redistribution (Os02t0795200) (Table 2). Auxin pathway genes control the cell elongation, root architecture, organ size, polarity and cell division. Shade responses include height elongation growth, increase in organ size like flag leaf, root and decrease in panicle length, which were also demonstrated in this study. Up-regulation of the above said genes supported the positive regulation of these auxin pathway genes for shade tolerance in SP. Earlier report of reduction of stem mechanical strength and redistribution of lignin^24^ indicated an involvement of asymmetric division and redistribution of auxin during low light stress. Present up-regulation of Os02t0795200 to ~1.8 fold (Table 2) supported the above indicated involvement of auxin in asymmetric division in shade grown panicles of SP. Down-regulated genes identified in this study included several auxin responsive factors such as *ARF10*, *ARF14*, *ARF13* and IAA amino acid hydrolase *ILR1-like 8*. ARFs bind to auxin response DNA element (AuxRE) to either activate or repress the transcription of auxin regulated genes and have been implicated in various abiotic stress responses. Their down-regulation in shade grown panicles require further analysis. The SAUR20 is a short-lived auxin-responsive gene which was earlier reported to be consistently expressed after temperature treatment in a Phytochrome interactive factor 4 (PIF4) dependent manner^25^. The down-regulation of *SAUR20* to ~1.44 fold (Fig. 7b) not only supported the earlier report as the shade treatment was associated lowered temperature, but also indicated the involvement of PIF4 in this response. In contrast, SAUR20 was up-regulated to ~5 fold (Fig. 7b) in N22 indicated some negative feedback of PIF4 in the panicle samples from shade.

### Light and clock regulation

All except 2 genes identified in the light signaling pathway were up-regulated in the shade grown panicles of SP in the present study (Table 2, Box-7 and Box-8). The up-regulated genes included several BBX genes such as *BBX4*; *BBX25*; *BBX22*; *BBX5*, *BBX19* and *BBX20*, the UV-B photoreceptor *UVR8*, the two phototropin genes *BTBN22* and *BTBN20*, several genes involved in flowering pathway such as *FLOWERING LOCUS T* (*FLT6*); *FLOWERING PROMOTING FACTOR1- LIKE PROTEIN-2* and the *MOTHER OF FLOWERING TIME* (*MFT1*), several CONSTANS-like (COL) genes such as *CCT35*, *COL4*, *COL5* and *COL3*. Expression of *CONSTANS* have been implicated as a master regulator of photoperiodic flowering^26^. *BBX4* (*COL3*) is a positive regulator of photomorphogenesis regulated root growth^27^, and thus its up-regulation to ~1.92 fold (Table 2) indicated its involvement in low light induced increased root length observed in this study. FLT6 controls flowering in SD by negatively regulating the expression levels of FT-like genes to result earlier flowering^28^. It’s up-regulation to ~1.7 fold (Table 2) supported its involvement in the earlier flowering in SP-shade grown plants. Up-regulation of the two phototropins in this study to ~1.5 and ~1.4 fold (Table 2) indicated their possible involvement in regulation of the blue light perception and signaling in the shade grown SP plants. CCT domain containing genes in rice were identified to regulate heading date. CCT35 (CMF11) is the evolutionarily closest to GHD7 that controls the grain number, height and heading date. Up-regulation of *CMF11* to ~3.3 fold (Fig. 7b) in the present study indicated its involvement to result earlier flowering, increased height and higher grain yield in the shade grown SP plants. Surprisingly, the *CCT35* expression in N22 samples were down-regulated to ~0.081 fold (Fig. 7b). This contrasting expression of *CCT35* could be the reason of low yield of N22 in shade compared to SP. The up-regulation of *UVR8* in the shade grown panicles could be due to some increased proportion of UV-B in shade, and requires further investigations such as detailed spectral analysis. Up-regulation of the *SOL1* (suppressor of LAZY1, causing the spreading phenotype of plants) indicated its involvement in having more erect stature of the plant including panicles in the present study. Up-regulation of several repressors of light and flowering pathway genes such as *BBX25* (*STH1*) and *COL4* (*BBX5*) indicate some feedback mechanism, and require further investigations. MOTHER OF FLOWERING TIME, MFT1, controls increase in spikelets per panicle and delay in heading date by suppression of EHD1, FZP and SEPALLATA-like genes^29^. The up-regulation of *MFT1* in SP shade grown panicles to ~11.3 fold and its contrasting down-regulation in N22 shade grown panicles to ~0.5 fold (Fig. 7b) strongly supported the basis of higher yield in SP in shade than N22. The up-regulation of *SPA1-RELATED 4* in shade grown panicles of SP (Table 2) indicated the involvement of Phytochrome A for manifestation of the low-light-grown-phenotype in mature plants of SP. The up-regulation of PHYA in SAS through FR mediated high irradiance response (HIR) has been reported in *Arabidopsis*^30^. Present up-regulation of *SPA1-RELATED 4* suggested that it might be involved to maintain constant levels of PHYA, required for HIR mediated shade tolerance response in SP. The two down-regulated genes identified under light signaling pathway were the *FLOWERING LOCUS K HOMOLOGY* (*PEP*) which is the positive regulator of floral repressor FLC and the negative regulator of light signaling *BBX19*. Up-regulation of *CCA1* and both the splice forms of *LHY1* to ~4.3 (Fig. 7b), ~2.2 and ~2 fold respectively in SP (Table 2) indicated involvement of clock in the shade tolerance response. A recent report demonstrated that higher levels of LHY/CCA1 protein is required for acclimatization of plants in cold stress^31^. Antagonistic down-regulation of CCA1 in N22 at ~0.05 fold might explain the shade tolerance in case of SP (Fig. 7b). BBX29, the Double B-Box 1 (DDB1) is a Heading Date 1 (HD1)-like protein, shown to have higher expression in seedlings, young panicles and stamens^32^. *BBX29* in this study showed contrasting expression of ~1.5 fold up-regulation in SP and ~0.4 fold down-regulation in N22 (Fig. 7b) indicated opposite regulation of this gene in the shade tolerance response in the above two genotypes.

### Photosynthesis related genes in shade tolerance response

Genes in the category of photosynthesis and carbohydrate metabolism included several candidates from components of photosystem II, chloroplast located genes, light harvesting complex, components of oxygen evolving system and phosphate uptake, homeostasis, sugar transporters (Table 2, BOX-5). The up-regulation of these genes indicated for increased chlorophyll content, increased oxygen evolution, net photosynthesis and increased photoassimilates. Up-regulation of sugar transporters such as *SWEET2B*, *SWEET16* and *SWEET13* (Table 2) indicated increased transport of photoassimilates from leaves to the panicles in the early stage of grain filling. Increased total sugar content in the SP-shade grown leaves in this study also supported the above observation of increased photoassimilates. Down-regulation of soluble starch synthase-2 *SSII-3* in the shade grown SP panicles (Table 2) indicated decreased partitioning of starch in the developing grains. This could be due to decreased conversion of sugar to starch due to low-light. Low light associated decreased starch accumulation, starch synthase activity and decreased amylose accumulation has been previously reported^33^. Up-regulation of photosystem II components such as *SGR*, *PSBS2* and *PSBP*s (Table 2) indicated increased synthesis of PSII components due to increased non-photochemical quenching and ROS production due to low-light stress, which has been reported in recent studies^34^. Down-regulation of the two monosaccharide transporters *MST1* and *MST7* (Table 2) indicated decreased cell wall synthesis^35^ and decreased sugar partitioning in the stamens^36^ respectively in shade grown panicles of SP.

### Genes involved in abiotic stress tolerance

In this category, 3/4^th^ of the genes were up-regulated, including several heat shock proteins (*HSPs*), heat stress transcription factors, heat shock factors (*HSFs*) genes responding to drought, low-temperature induced protein, genes involved in repair of photosystem II and genes in iron metabolism, whereas the 1/4^th^ of the down-regulated genes included 11 s globulin seed storage protein, fructokinase-2, Phosphoenolpyruvate kinase (*PEPC*), gene from salicylic acid pathway, HSF and senescence specific cysteine peptidase (Table 2, BOX-6). These observations indicated a major involvement of HSPs and HSFs for manifestation of shade tolerance responses.

### Genes controlling panicle length

For further evaluation of contrasting panicle emergence phenotype in SP and N22, real-time PCR for two panicle length controlling genes (Long Panicle; LP1 and Short Panicle; SP1) was performed. LP1 represented either a superior or an inferior allele for having either long or short panicles respectively in recent reports^37^. In the present study, *LP1* was down-regulated to ~0.71 fold in SP, whereas up-regulated to ~2.1 fold in N22 (Fig. 7c). This observation indicated that N22 could have the inferior allele of *lp1*. However, the panicle length of SP and N22 were nearly equal in sun (Fig. 3) and difference in PL in shade was due to the remarkable increase of PL, which was observed only SP (Fig. 2h). This observation ruled out the possibility of LP1 to be the reason of contrasting difference of PL between SP and N22 in the present study. SP1 is responsible for the meristematic activity in inflorescence and thereby controlling inflorescence branch elongation. SP1 is reported as a nitrate transporter highly expressed in the phloem of the branches of young panicle^38^. *SP1* expression was up-regulated to ~1.5 fold in SP whereas down-regulated to 0.6 fold in N22 (Fig. 7c). This contrasting *SP1* levels in SP vs. N22 could explain that lower levels of *SP1* in N22 panicles in shade restricted the panicle length elongation in shade with the occurrence of its opposite phenomenon in the SP panicles.

## Discussion

The present study was conducted with several aims to understand the shade tolerance responses and its effects on crop yield. Briefly, these aims included to identify seedling phenotypes, mature plant phenotypes, physiological characteristics, biochemical parameters and the genes involved or affected by shade or low light stress. From the three different durations of rice used for this study, it was concluded that short duration rice such as Nagina 22 and Swarnaprabha (SP) were better suited to be grown under shade than the medium or long duration rice such as KMR3 or Swarna respectively. This interpretation was based on the tolerant mature plant phenotype of N22 and SP in shade. This could be due the fact that short duration rice gets a smaller number of days in shade compared to the long duration rice to complete their life cycle. While intermediate coleoptile length was identified as the characteristic seedling phenotype in a short period screening of 8 days on petriplates, longer shoot length and higher rootlet number were demonstrated to be the characteristic phenotypes in a longer period of screening for 28 days in seedlings grown in hydroponic medium. However, these seedling phenotypes did not correlate with the shade tolerance in terms of yield, as sustainable yield in shade was obtained only in SP with least decrease in yield/plant (17.3%) among all the lines tested. Yield/plant in shade correlated significantly with only 3 parameters out of 21 traits tested, which were NDVI index, panicle length and grain filling percentage. SP showed the least % decrease in yield/plant in shade followed by the IL of Swarna 192S with 33.2% decrease. The wild introgressions from *O*.*nivara* in 192S^12^ could be the reason of sustainable yield in shade and showed importance in agronomic point of view. Among the 6 biochemical traits tested, only the decrease in the superoxide dismutase (SOD) activity correlated with high yield in sun and shade. Study of carbon, nitrogen and phosphate partitioning interpreted that higher % of carbon was essential for a shade tolerant plant in term of yield. This higher carbon % could be imparted to up-regulation of sugar transporters (SWEETs) in the shade grown panicles of SP indicated increased bi-directional sugar transport across the cell membrane and also increased pathogen resistance, senescence, for which the SWEET transporters are known^39^. However, down-regulation of two monosaccharide sugar transporters (MSTs) and the soluble starch synthase *SSII-3* indicated some negative feedback of carbon resource allocation along with decreased conversion of sugar to starch in grain in SP shade grown panicles. Gene expressions in R and low R/FR in the seedling stage for the two major gene involved in SAS (i.e PHYB and PIL13) revealed interesting results. The expression patterns of *PHYB* and *PIL13* in the shade grown panicles were reverse in SP and N22 in shade could be associated with shade tolerance response in SP. The decreased *PHYB* and increased *PIL13* expressions under low R/FR in SP was in agreement with previous reports in *Arabiodopsis*^15^, which indicated that the tolerance response to prolonged low R/FR in SP could be similar to that in *Arabidopsis*. However, the opposite pattern of expressions of *PHYB* and *PIL13* under cR and low R/FR in N22 indicated variation in the mode of function of PHYB and PIL13. These observations of different modes of action in N22 might be linked to its being a drought and heat tolerance variety^40^ accommodating high light and heat environments, however requires further investigations. This observation was further supported by the results of up-regulation of *SAUR20*, which functions at high temperature in a PIF4-dependent manner^25^, in N22 shade grown panicles to ~5 fold. Genome-wide expression analysis revealed that most of the ethylene and cytokinin pathway genes were up-regulated indicating that these were the major positive regulators for shade tolerance responses in rice. While most of the genes identified in light and clock signaling were up-regulated, auxin pathway and photosynthesis related genes showed a major proportion among the up-regulated with minor proportion among the down-regulated category. Most of the genes selected for real-time PCR showed characteristic gene expression in SP shade grown panicles which significantly differed in N22. Owing to the higher contrasting differences in the expression levels between SP and N22, expressions of *MFT1*, *EREPB*-*2* were notable. Despite ~11-fold up-regulation of *MFT1*, a flowering repressor, the flowering time in SP in shade was not delayed. It could be due to the fact that the effect of *MFT1* in delaying the flowering time was counter-acted by the up-regulation of several other regulators of flowering such as *FLT6*, *CMF11* in this study. Up-regulation of Short panicle (*SP1*) expression in SP and its corresponding down-regulation in N22 panicles indicated that SP1 could be involved in increase of panicle length in shade in case of Swarnaprabha. Lastly, we detected the two splice forms of PHYB (Os03t0309200-01 and Os03t0309200-02), which has been presented in Rice Annotation Project Database (RAP-DB), at ~0.32 and ~0.13 fold respectively in in the complete data (https://www.ncbi.nlm.nih.gov/geo/query/acc.cgi?acc=GSE118464). The detection of the splice variants of PHYB encompassing different exon intron gene structure in the shade grown panicles indicated a yet unknown functional relevance in shade tolerance that requires further study.

## Conclusion

It could be concluded that short duration rice (Swarnaprabha in this study) is more suited for shade tolerance response. Intermediate coleoptile length and higher rootlet number were identified as discriminating seedling characteristics grown under low R/FR. Lower superoxide dismutase activity in shade was the only biochemical parameter associated with shade tolerance. Increase in panicle length with increased percentage in grain filling in shade were demonstrated as the noble mature plant phenotypes necessary for sustainable yield. Up-regulation of *COL3* could be associated with longer root length in seedlings under low red/far-red. Shade tolerance in Swarnaprabha could be assorted to significant up-regulation of *MOTHER OF FLOWERING TIME1* (*MFT1*) along with *ETHYLENE RESPONSE ELEMENT BINDING PROTEIN-2* (*EREBP-2*), and *SHORT PANICLE1* (*SP1*) in the shade grown panicles. The drastic up-regulation of *MFT1* and *EREPB-2* in the shade grown panicles of Swarnaprabha can be used as important clues to understand shade tolerance response. The association of panicle length with tolerance to shade in this study was the noble finding till date.

## Materials and Methods

### Plant growth conditions

Rice seeds were imbibed on wet blotting paper and incubated at room temperature in dark for 48 hours (h) followed by 8 h of germination induction under white light (WL). Seedlings of 15-days old were transferred to pooled pots and grown for 1 months, after which they were transferred to single plant per pot. Plants were kept in sun or under continuous shade 55 days after sowing (DAS). Plants were grown in 12-inch pots containing 8 L soil from rice fields with urea (1 g), potassium (200 mg) and phosphate (2 g). Urea was applied thrice (during soil preparation, in growth stage and prior to booting stage) to the plant. The study was done during Apr 2017– Sep 2017 (season 1) and Sep 2017 to Apr 2018 (season 2). The bright sun light in season 1 varied within the range of maximum 1351.8 μmols m^−2^ sec^−1^ and minimum 839.2 μmols m^−2^ sec^−1^ and that in season 2 varied within the range of maximum 1325.8 μmols m^−2^ sec^−1^ and minimum 310.3 μmols m^−2^ sec^−1^. The geographic location of NISER is at an altitude of 38 meters above the sea level with latitude/longitude: 20°09′35″N85°42′26″E. The temperature, humidity (RH) and rainfall (RF) as recorded by The Metrological Department situated at Jatni station was average 43 °C max, 34 °C min, 82.7% RH, 177.2 mm RF respectively in the season 1and that in season 2 was average 41 °C max, 30 °C min, 71.5% RH, 23 mm RF respectively.

### Seedling phenotype

Seedlings were grown on petriplates for 7 days under either continuous darkness (D), continuous red (cR) or low red/Far-red (cR/FR) after the WL germination induction (Supplementary Fig. 1). The WL was obtained from Philips17 W F17T8/TL741 USA Alto II technology tubes fitted in plant growth chambers (Model-AR36, Percival, USA), set to 22 °C and 70% relative humidity. WL obtained was with 50% light intensity, which is equivalent to ~100 μmols m^−2^ sec^−1^. For monochromatic red (R), red/far-red (R/FR) light irradiations, seedlings were grown in E-30 LED chambers (Percival, USA) maintained at 22 °C temperature and with 70% RH. The intensity of incident light was 50 μmols m^−2^ sec^−1^ for R. For low R/FR light, both the R and FR lights were illuminated and adjusted so as to get the R/FR ratio to 0.06. For Hydroponic experiment, seedlings were grown for 10 days on petriplates and then transferred to medium containing half strength of Hoagland’s solution^41^ for 15 days till the phenotyping was done. Seedlings were grown in 6.4 cm × 6.4 cm × 10 cm planton tissue culture containers (Tarsons Product Pvt. Ltd. India) with 250 ml of Hoagland’s media. For seedling phenotype analysis, shoot length, root length, rootlet number and coleoptile length was measured from 25–30 seedlings in 2 independent biological replicates. For significance analysis, one-way ANOVA with Bonferonni’s multiple comparison was performed using Prism version 7.0 from the selected lines under specific light condition. The comparison of the data values from D was compared with that of R, similarly data values from R was compared with that of R/FR. Statistically significant difference (p < 0.01) is indicated as **** in graph.

### Mature plant phenotype

All mature plant phenotype was recorded from plants grown in open field inside net house situated at NISER (Supplementary Fig. 2) in completely randomized block design in 2 replications. Shade was obtained by covering the net house with 75% quantified cutoff agro net from B&W Agro Irrigation Co Mumbai. Mature plant phenotype was recorded as previously^12^. Briefly, NDVI index and quantum yield (Qy) were taken 15 days after transfer into shade or sun. Plant height (PH), number of productive tillers (NPT), relative water content (% RWC), internode length (INL) and flag leaf length (FLL) were recorded 15 days after panicle emergence. All biochemical assays (i.e. enzymatic assays, total sugar, carotenoid, and flavonoid content) were analyzed from flag leaves 15 days after panicle emergence. For statistical significance, one-way ANOVA with Bonferonni’s multiple comparison was performed using Prism version 7.0.

### Enzyme activity assay

Superoxide dismutase (SOD), catalase and peroxidase enzyme activity were determined spectrophotometrically as described by Dhindsa *et al*.^42^, Aebi^43^ and Castillo *et al*.^44^ respectively. For these assays, 100 mg of leaf sample (flag leaf) was ground with 4 ml of extraction buffer (0.1 M phosphate buffer, pH 7.5, containing 0.5 mM EDTA) and filtered through 4 layers of cheese cloth. The filtrate was transferred to centrifuge tubes and centrifuged at 15000 rcf for 20 min. The supernatant was used as the enzyme extract and 100 μl of this extract was used for each enzyme activity assay. Briefly, SOD activity was determined by measuring the decrease in the absorbance of blue colored formazone and ${{\rm{O}}}_{2}^{\cdot -}$ at 560 nm. For determining catalase activity, the reaction was started by adding H_2_O_2_ (12.5 mM) to the enzyme extract and decrease in the absorbance was measured at 240 nm for 1 min. Peroxidase activity was determined by increase in the optical density due to oxidation of guaiacol to tetra-guaiacol in the reaction mixture for 2 min, measured at 470 nm.

### Total sugar content

Total sugar content was estimated using phenol-sulphuric acid method as explained by Buysse *et al*.^45^ with small modification^12^. 100 mg leaf sample (flag leaf) was boiled twice consecutively in 5 ml of 80% methanol for 5 min each time. Supernatant was collected and pooled from the two steps. To 1 ml of this supernatant, 1 ml of 18% phenol and 5 ml of concentrated H_2_SO_4_ was added. The mixture was vortexed vigorously for 10 min. Amount of total soluble sugar in the plant leaf samples were determined using glucose: fructose: galactose (1:1:1 v/v) standard curve. The absorbance was measured as 490 nm.

### Carotenoid content

Twenty-five mg of leaf sample was frozen in liquid nitrogen and extraction of total carotenoid was done in 3 ml 80% acetone extracts. The ground samples were kept in dark in 4 °C for 48 h. Estimation of carotenoids was done in the supernatant after centrifugation at 10,000 rpm at 4 °C for 10 min according to Arnon^46^ by using the following formula

$${\rm{Carotenoid}}\,(\mathrm{ug}/\mathrm{mg})=(({\rm{A}}480+(0.114\ast {\rm{A}}663)-(0.638\ast {\rm{A}}647))\ast \,3000)/(1000\ast FW)$$

### Flavonoid content

Total flavonoid content was determined as according to Harborne^47^. The flavonoid content in the seedlings was estimated using HCl-chloroform method. Briefly, 50 mg of leaf samples (flag leaf) were ground with liquid nitrogen. To the frozen powder, 1.5 ml of 1% HCl (w/v) in methanol was added and kept at room temperature for 2 h. 1 ml of chloroform was added to it and centrifuged at 7000 rpm for 10 min at room temperature (RT). The flavonoid content was determined spectrophotometrically by measuring the absorbance at 300 nm.

### Carbon, nitrogen and phosphorus estimation

Estimation of nitrogen in the flag leaf and panicle samples were done using Micro-Kjeldahl method^48^. Estimation of phosphorus was done using Vanado-molybdate yellow-color method^49^. Estimation of carbon was done using an element analyzer^50^. For all these estimations, flag leaf and panicle samples sampled at final stages of grain filling (~15–21 days after panicle emergence) were dried, ground to powder form and 0.5 g of the dried samples was used for estimation. Briefly, in Micro-Kjeldahl method of nitrogen estimation, dried plant sample was digested at 410 °C in 10 ml of sulphuric acid with copper sulphate as catalyst. The digested sample was distilled in the Kjeltec 1026 distilling unit and mixed with 20 ml of 4% boric acid along with 3–4 drops of indicator. The distillate collected was titrated with 0.1 N H_2_SO_4_ and the titre value was noted and calculated in to % nitrogen per fixed wt. For phosphorus estimation, cold digestion of the dried plant sample was done in triple acid mixture (concentrated nitric acid, perchloric acid and sulphuric acid in the ratio 10:4:1) for overnight followed by digestion at 200 °C for 2 h. The digest was made up to 100 ml. To 5 ml of the digest, 5 ml of vanado-molybdate reagent was added followed by making the volume to 25 ml with distilled water. After 10 minutes absorbance was measured at 490 nm. For estimation of carbon ground plant samples were made to pass a 0.15 mm sieve.

### RNA isolation and Gene expression study

Total RNA was extracted from seedling samples irradiated with 7 days red (cR), low red/far-red (cR/FR) or kept at dark (D) using TRIzol Reagent (Invitrogen™ 15596018) and quantified using Nanodrop. Four µg of RNA were treated with DNase (New England Biolabs M0303s) followed by a purification step using sodium acetate (NaOAC). Briefly, RNA was mixed with 1/10^th^ v/v of 3 M NaOAC and 1/4^th^ v/v absolute ethanol. It was frozen in liquid N_2_ for 5 min followed by centrifugation at 12000 rpm for 5 min at 4 °C. The samples were washed with 75% ethanol and eluted with 10 μl of RNase-free water. For the semi-quantitative RT-PCR, relative expression level was calculated using Image lab software (Version 6.0.0, 2017, Bio-rad Laboratories). The cDNA was prepared using Bio-Rad iScript™ Reverse Transcription Super-mix, (Cat #1708840). The quantitative RT-PCR was performed using CFX384 Touch™ Real time detection system (Bio-Rad laboratories), following the manufacturer’s manual of iTaq™ universal SYBR green supermix. Gene-specific primers used for qRT-PCR are listed in Supplementary Table 3. The PCR recipe and the thermocycler program used were similar to as done previously^51^. For normalization *ACTIN* was used as control. Each qRT-PCR reaction was performed in at least triplicate and all data were presented as mean ± SEM. Primers used for the gene expression study (PHYB, PIL13 and PIL14) are listed in Supplementary Table 3.

### Microarray analysis

Panicles on the day of complete emergence from SP sun and shade treated plants were sampled, spikelets were separated from the main shoot quickly in the same light condition and immediately frozen in liquid nitrogen. Two panicles from the mother tiller either from sun or shade treated plants were sampled separately from two individual plants. Experiment was performed in duplicates. Total RNA was extracted from these panicle samples using Qiagen Rneasy Mini Kit (Cat. No. 74004). The concentration and purity of the RNA evaluated using the Nanodrop Spectrophotometer (Thermo Scientific; 1000). The integrity of the extracted RNA were analysed on the Bioanalyzer (Agilent, 2100). The microarray hybridization and scanning were performed at the Agilent certified microarray facility of Genotypic Technology, Bengaluru, India. Hybridization was done on Rice Agilent 4 × 44 K array (AMADID: 064815). Hybridization was performed in arrays containing 42249 probe sets according to the Agilent GeneChip expression analysis technical manual using Agilent Technologies, *In situ* Hybridization kit, Part Number 5190-0404. Raw data was analyzed using Agilent Gene Spring GX software. For differential expression analysis, genes showing >/< 0.6 fold change (FC) were selected as up or down regulated respectively, where FC was calculated in log base 2. Statistical student T-test p-value among the replicates was calculated based on volcano Plot Algorithm. Differentially regulated genes were clustered using hierarchical clustering based on Pearson coefficient correlation algorithm to identify significant gene expression patterns. Biological analysis was performed for the differentially expressed genes based on their functional category and pathways using Biological Analysis tool DAVID (http://david.abcc.ncifcrf.gov/). Higher p-value were included in this analysis to include majority of genes of interest required to understand the response to shade tolerance response. Microarray data has been submitted in NCBI repository and can be accessed at the GEO accession server at https://www.ncbi.nlm.nih.gov/geo/query/acc.cgi?acc=GSE118464.

### Real-time PCR (Q-PCR) analysis

RNA extraction, quantifying and quality check for Q-PCR was similar to that in microarray. The primers were designed for selected genes using Primer 3 Plus online primer design software considering exonic and coding region of the transcripts. Specificity was verified using In-Silico PCR in UCSC *In*-*Silico* PCR online bio-informatics tool. Primers were validated for their specificity using pooled cDNA from all the samples. The total RNA were converted into cDNA using Affinity Script qPCR cDNA synthesis kit (Agilent Technologies, USA). Primers used in the Q-PCR are listed in Supplementary Table 3. SYBR Green chemistry (Brilliant II SYBR Green qPCR master mix (Agilent Technologies, USA) in Stratagene mx3005P instrument (Agilent Technologies, USA). The amplification cycling conditions are as follows; Initial denaturation for 95 °C for 10 min followed by 40 cycles of 95 °C for 30 sec, 60 °C for 30 sec. The relative quantification of genes was analyzed using standard 2^−ΔΔCt^ as described by Pfaffl^52^. Primers used for microarray data validation are listed in Supplementary Table 3 .

## Supplementary information

Supplementary File Online

## Data Availability

The datasets generated during and analyzed during the current study are available in the NCBI repository at the GEO accession server, https://www.ncbi.nlm.nih.gov/geo/query/acc.cgi?acc=GSE118464. All data analyzed during this study are included in this article and its Supplementary online Material.
