# Supplementary material for: Shade tolerance in Swarnaprabha rice is associated with higher rate of panicle emergence and positively regulated by genes of ethylene and cytokinin pathway
Source: Sci Rep. 2019 May 2;9:6817. doi: 10.1038/s41598-019-43096-8 (PMC6497668; doi:10.1038/s41598-019-43096-8)
Supplement: Supplementary file 1 — Supplementary File Online [file 41598_2019_43096_MOESM1_ESM.pdf]

Shade tolerance in Swarnaprabha rice is associated with higher rate of panicle emergence and positively regulated by genes of ethylene and cytokinin pathway

Madhusmita Panigrahy[1]*, Aman Ranga[1], Jyotirmayee Das[2], Kishore CS Panigrahi[1]*

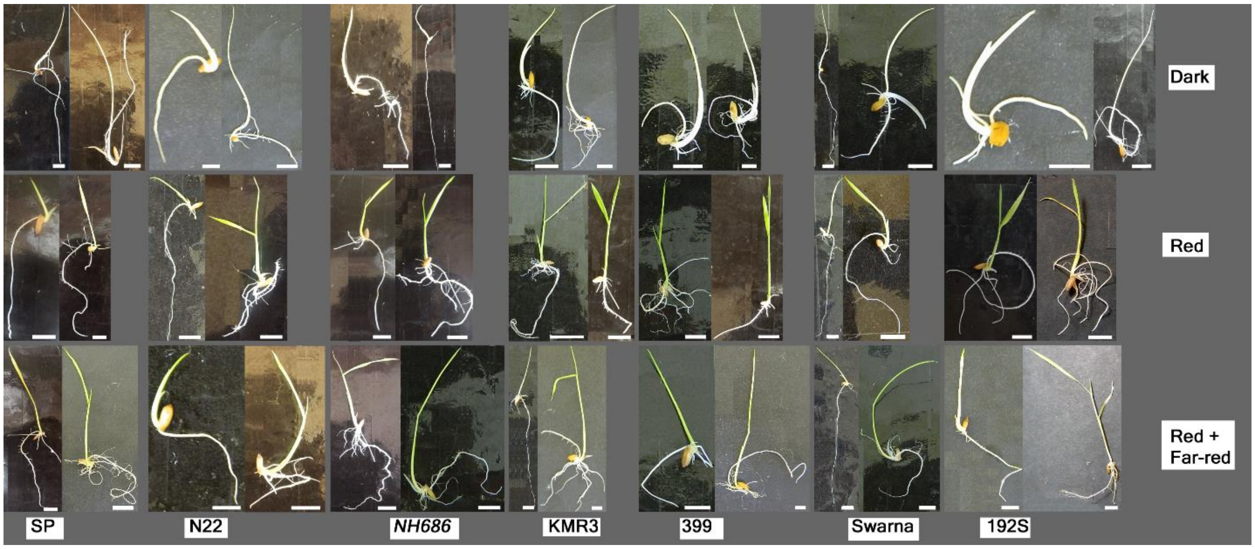

Supplementary Figure 1. Representative images of seedlings of 7 rice genotypes grown in different light quality. Seedlings of SP, N22, KMR3, Swarna, the mutant of N22 *NH686*, the IL of KMR3 399 and the IL of Swarna 192S were grown for 7 days either on petriplates or in hydroponic medium till 25 days in complete Darkness, continuous Red (cR) or low cR/FR. One representative seedling from each genotype from every light condition from petriplates or hydroponic medium were photographed and presented. 1st, 2nd and 3rd row in the figure are the seedlings grown in Dark, Red or low cR/FR respectively. In each sub panel the 1st seedling was grown in petriplates and the 2nd seedling was grown in hydroponic medium respectively. Scale bar: 5 cm.

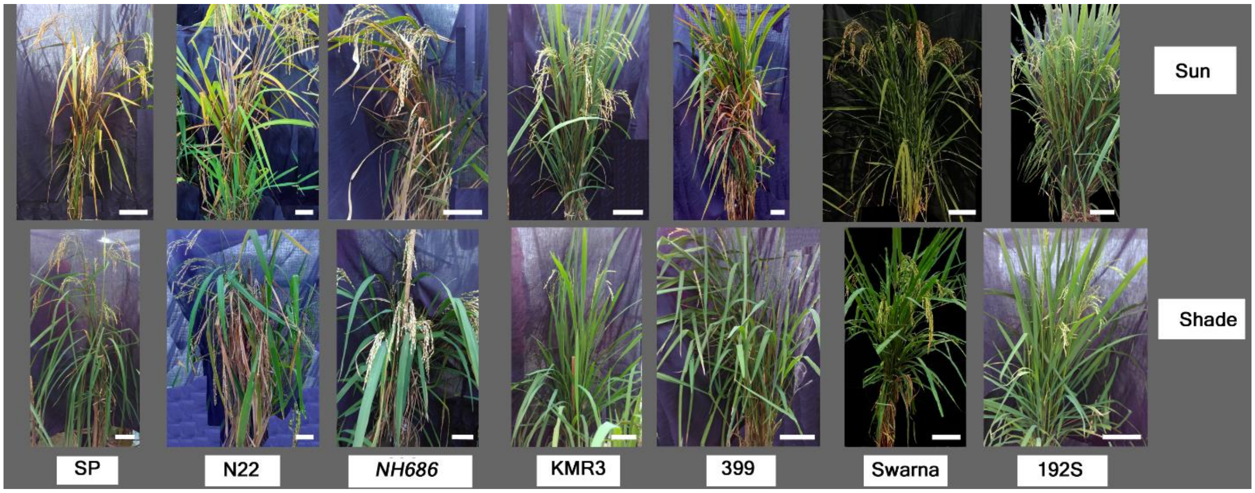

Supplementary Figure 2. Pictures of mature plants photographs of 7 rice genotypes grown under continuous sun or shade conditions. Seven genotypes (SP, N22, KMR3, Swarna, the mutant of N22 *NH686*, the IL of KMR3 399 and the IL of Swarna 192S) were photographed 15 days after day to flowering. Top and bottom row shows the plants grown under sun or shade condition respectively. Scale bar: 5 inch.

Supplementary Table 1. Percentage increase or decrease in the mature plant morphological and yield related traits in shade. Percentage increase or decrease of mature plant morphological and yield related traits were calculated from the data values (Figure 2 and Figure 3) of sun vs. shade. NPT: total number of productive tillers, DBM: dry weight of above ground biomass, NDVI: normalized difference vegetation index, Qy: quantum yield, RWC: % relative water content, INL: length of the 1st internode, PL: length of the panicle after harvest, FLL: length of the flag leaf, Flav: flavonoid content, Car: carotenoid content, SOD: superoxide dismutase enzyme activity, CAT: catalase enzyme activity, POX: peroxide enzyme activity, 100-Gwt: grain weight of 100 filled grains, %GF: percentage grain filling, Gwt/pa: weight of filled grain in a panicle, Yi/Pl: yield per plant.

	Height (% Increase)	NPT (% decrease	DBM (% decrease	NDVI (% Increase)	QY (% Increase)	RWC (% Increase	FLL (% increase)	INL (% decrease)	PL (% decrease)	Flav (% increase)	Car (% decrease)	Sugar (% increase)	SOD (% increase)	CAT (% increase)	POX (% decrease	1000-Gwt (% decrease	%GF (% decrease	Gwt/Pa (% decrease	Yi/Pl (% decrease)
**SP**	6.30	47.06	51.01	0.90	4.52	5.04	32.83538	5.07	-25.22	14.95	29.80	46.57	3.60	27.84	27.95	10.70	-40.23	-16.59	17.39
**N22**	15.38	-27.03	-6.77	3.82	13.46	-5.60	23.34253	22.57	27.46	5.90	57.37	-139.70	19.95	-15.59	32.53	28.13	14.85	72.41	74.18
NH686	4.19	61.54	27.66	4.72	18.25	23.01	16.3228	12.47	28.68	-74.00	-3.04	-227.66	-6.57	43.08	51.01	7.81	8.11	53.37	75.28
**KMR3**	-34.91	45.45	65.39	4.99	5.41	-13.84	16.12984	24.57	25.20	-27.46	48.52	33.55	26.99	21.33	14.59	-2.66	0.71	48.55	69.64
**399**	-15.20	45.45	80.79	2.91	2.65	-2.21	-40.8171	8.96	25.67	-8.47	-8.62	49.94	37.54	73.23	30.97	22.14	1.07	66.11	88.15
**Swarna**	-7.70	55.86	57.77	1.45	1.39	12.40	1.538034	9.57	13.29	-23.59	-3.33	23.91	1.40	8.06	14.49	21.60	28.45	-10.10	49.37
**192S**	-16.00	65.74	64.30	3.06	3.87	-9.79	-3.3583	10.60	0.62	17.17	-85.03	-3.12	60.81	30.90	37.30	-4.67	-18.04	29.60	33.29

Supplementary Table 2. Correlation of percentage decrease in Yield/plant with other mature plant parameter or biochemical characteristics. * and ** indicate significant correlation at $p<=0.05$ and $p<=0.01$. Pearson's correlation coefficient $r= 0.666$ at $p<=0.05$ and $r= 0.798$ at $p<=0.01$.

	**Yi/Pl**
**PH**	-0.126
**NPT**	-0.319
**DBM**	-0.146
**NDVI**	**0.676***
**QY**	0.389
**RWC**	0.004
**FLL**	-0.444
**PL**	**0.942****
**INL**	0.506
**1000-Gwt**	0.362
**%GF**	0.652
**Gwt/Pa**	**0.846****
**Yi/Pl**	1.000
**Flav**	-0.524
**Car**	0.285
**sugar**	-0.375
**SOD**	-0.009
**CAT**	0.223
**POX**	0.148

Supplementary Table 3. List of Primers used for Gene Expression and real-time PCR analysis

SI.No	Primer ID	Primer Seq 5' to 3'
1	ACO1_FP	GCAGATCGACGGCAACAGGA
	ACO1_RP	TGGCGCACGTACAGCTTCAT
2	CCA1 _FP	GAGGTGCTCAGTGGAAGCGA
	CCA1 _RP	TCTGACTTGGTAGAGGCCAGCA
3	CKX11_FP	TTTGCGTGTGACAGGTGGGA
	CKX11_RP	TTGTTCTGCGCCACCAGCTC
4	CP24_FP	CCGGCGGCAAGTTCTTCGAC
	CP24_RP	ATGAGCATGGCGAGCATGGC
5	ERS2_FP	GGGCTGGCCCTTTCCAGAAG
	ERS2_RP	GGCGCCATTCTTCGGAGGTT
6	LOGL3_FP	GCATCATCGTGTCGGCTCCA
	LOGL3_RP	AGCTCGCTGCAACAGCTTAGA
7	MFT1_FP	CGCCGCCCTCTACTTCAACG
	MFT1_RP	CATGGCCGACCGATGGCTTA
8	PHYB_FP	CCGCTGGACAACCCAAGAGG
	PHYB_RP	ACTTGCTGCTTGCTGAGGCT
9	SAUR20_FP	CCCGACGGACTACCTGAAGC
	SAUR20_RP	CTCGACGGCCTTGAGGATGG
10	SP1_FP	GCTCAGCGACAACGACCTCA
	SP1_RP	GCACGGTCGTCTCCACACTC
11	SSII-3_FP	CCGCCGAGCTCTACGAGGA
	SSII-3_RP	AGCAGCGCCAAGCTTCTTCA
12	TIFY11B_FP	CATCATGAGGAAGGCGTCGCT
	TIFY11B_RP	GGCGAGGTCCAGCCATGAG
13	PIL13_FP	GGGCCTCAAGTAGCACAGCA
	PIL13_RP	CGACCCTTTGTGCAAAGCCTACA
14	PL1_FP	GGAACACCCTCAGGTCTGCAC
	PL1_RP	TGGCAAGTGAAGCAGCCACT
15	EREBP-2_FP	ACAAGCCCAAGCCACCACTC
	EREBP-2_RP	GCAACAATCAGAGCATGGAACCG
16	BBX29_FP	TCTCCAGGAGTGGTGCCAAC
	BBX29_RP	GCCTGGCTGAAGACCAAAGC
17	CCT35_FP	GCAAGGCCTACGCCGAGATG
	CCT35_RP	CGGAACCATCCGAGGTCGAG
18	OsPIL14_FP	ACGACGACGACGACATCG
	OsPIL14_RP	GCTTGAACAGCGGGTAGC
19	Actin_FP	GATCACTGCCCTCGCACCAA
	Actin_RP	TTGCTGGACCCGACTCATCA
